# Acute skin toxicity and cosmesis outcome in non-metastatic breast cancer patients treated with ultrahypofractionated radiotherapy: a randomized controlled phase II clinical trial comparing proton versus photon radiotherapy

**DOI:** 10.1093/jrr/rrag037

**Published:** 2026-05-25

**Authors:** Thanaporn Sarsitthithum, Chonnipa Nantavithya, Chawalit Lertbutsayanukul, Kitwadee Saksornchai

**Affiliations:** Division of Radiation Oncology, Department of Radiology, King Chulalongkorn Memorial Hospital, RamaIV Road, Pathum Wan, Bangkok 10330, Thailand; Division of Radiation Oncology, Department of Radiology, King Chulalongkorn Memorial Hospital, RamaIV Road, Pathum Wan, Bangkok 10330, Thailand; Division of Radiation Oncology, Department of Radiology, Faculty of Medicine, Chulalongkorn University, RamaIV Road, Pathum Wan, Bangkok 10330, Thailand; Division of Radiation Oncology, Department of Radiology, Faculty of Medicine, Chulalongkorn University, RamaIV Road, Pathum Wan, Bangkok 10330, Thailand; Division of Radiation Oncology, Department of Radiology, Faculty of Medicine, Chulalongkorn University, RamaIV Road, Pathum Wan, Bangkok 10330, Thailand

**Keywords:** proton therapy, breast radiotherapy, hypofractionated, breast cancer

## Abstract

The 1-week photon radiotherapy schedule of 26 Gy in 5 fractions is as safe and effective as the standard 3-week schedule of 40 Gy in 15 fractions for adjuvant local radiotherapy in early stage breast cancer. Proton radiotherapy offers dosimetric advantages by reducing heart and lung dose but may be associated with increased acute skin toxicity, and the acute toxicity of ultra-hypofractionated proton therapy remains unknown. In this randomized trial, non-metastatic breast cancer patients were assigned to receive either proton or photon radiotherapy at a dose of 26 Gy in 5 fractions delivered on alternate days, with the aim of comparing acute skin toxicity between the two modalities. Of 140 eligible patients, 72 were included in this interim analysis, with 36 patients in each treatment arm. The highest observed toxicity was grade 2 radiation dermatitis, which occurred in one patient in the proton arm. The incidence of acute radiation dermatitis was significantly higher in the proton arm than in the photon arm (97.2% vs. 75%, *P* = 0.006), while no significant differences were observed in other acute toxicities. Patients treated with photon radiotherapy demonstrated better cosmetic outcomes (41.7% vs. 13.9%, *P* = 0.007) and higher satisfaction scores (80.6% vs. 58.3%, *P* = 0.04) compared with those treated with proton radiotherapy. At a median follow-up of 9 months (IQR: 6–11), no locoregional recurrence was observed in either treatment arm. This interim analysis indicates that ultra-hypofractionated proton therapy is associated with a higher incidence of mild acute radiation dermatitis, whereas photon radiotherapy results in superior cosmetic outcomes and patient satisfaction, with longer follow-up required to determine long-term outcomes.

## INTRODUCTION

Breast cancer is the most prevalent cancer in Thai women [1], and the standard treatment for early stage breast cancer comprises surgery, chemotherapy, targeted therapy and radiotherapy. Radiotherapy after breast-conserving surgery is crucial to reduce locoregional recurrence and breast cancer deaths [2, 3].

In King Chulalongkorn Memorial hospital, ~2000 new breast cancer patients have been treated at Division of Therapeutic Radiation and Oncology with conventional fractionation (45–50 Gy in 25 fractions of 1.8 or 2 Gy/day, 5 days a week, for 5 weeks) or hypofractionation (40.5–43.5 Gy in 15-fraction or 16-fraction of 2.65–2.7 Gy/day, 5 days a week, for 3 weeks) as recommended in international guidelines [4, 5].

However, ultrahypofractionation, a 1-week schedule with a total dose of 26 Gy in 5 fractions, has demonstrated non-inferior tumor control [6, 7] and provided several benefits in terms of patient convenience and cost reduction. Although concerns have been raised about late radiation toxicities due to the higher dose per fraction in ultrahypofractionation, the FAST-forward trial revealed that these toxicities were not inferior to those of hypofractionation [7].

Due to the Bragg peak [8], proton radiotherapy (PRT) has shown superior results in sparing heart and lung dose compared to photon therapy [9–11]. However, acute skin toxicities have been reported in early breast cancer patients receiving PRT through conventional fractionation [12]. When we compare the biological effective dose (BED) between conventional dose and ultrahypofractionated dose [7], the latter has lower value of BED (Appendix 1). Therefore, the acute responding tissue toxicity, i.e. skin, in ultrahypofractionated dose might not be as high as in conventional dose. If acute skin toxicity of ultrahypofractionated dose in PRT isn’t much higher than photon radiotherapy, using PRT will be beneficial for patients by sparing heart and lung dose, thus preventing late effects which can cause morbidity and mortality for patients.

Therefore, to our knowledge, this is the first randomized control trial aims to compare acute skin toxicity between PRT and photon beam radiotherapy using ultrahypofractionation in non-metastatic breast cancer patients.

## METHODS

### Study design and patients

This is a single-center, non-blinded, phase 2 prospective randomized study conducted at King Chulalongkorn Memorial Hospital in Thailand. The eligible patients were women aged at least 21 years with pathologically confirmed breast cancer (stage 1–3 according to AJCC 8th edition), a good performance status (ECOG 0-2), and who underwent mastectomy or lumpectomy with negative margins. Axillary lymph node dissection or sentinel lymph node biopsy was performed according to the initial nodal status. Exclusion criteria included patients with active skin problems on the chest wall and axillary area, connective tissue disease or other non-malignant systemic diseases that would prevent them from receiving study treatment or required follow-up, as well as pregnant or lactating women. Patients who had received radiation therapy for their currently diagnosed breast cancer prior to randomization, or prior radiation therapy to the ipsilateral chest wall or ipsilateral breast, as well as those who received bilateral whole breast or chest wall irradiation, were also excluded. The study protocol was approved by the Research Ethics Committee of the King Chulalongkorn Memorial Hospital (IRB number 017/64) and written informed consent was obtained from all patients. Patients who declined to take part in the study received the same standard of care as the patients who agreed to take part in the study.

### Randomization and masking

Patients were randomly assigned to receive either photon (control group) or proton therapy (intervention group) with the same dose of radiation by using the computer. Randomization was stratified by type of surgery (mastectomy or lumpectomy). Concealment was performed by a statistician and generated by the STATA program using the command ‘ralloc’ for random allocation of treatment. Treatment allocation was not masked to clinicians or patients due to different radiation machines.

### Radiotherapy

This trial adopted a radiation dose of 26 Gy in 5 fractions from the FAST-Forward trial (5.2 Gy/fx) [7]. Due to logistical constraints, we designed the radiation schedule to be given on alternate days. Patients with age < 50 years or high grade (≥Grade 3) or close margins (<2 mm) for both intervention and control groups received a tumor bed boost by using simultaneous integrated boost (SIB) techniques. This allowed physicists and physicians to use only one treatment plan. The SIB consisted of an additional 1 Gy/fx to the tumor bed with a total dose of 31 Gy for 5 fractions (6.2 Gy/fx). The SIB was given by proton in the experimental arm and photon in the control arm. In mastectomy patients who were randomized to the photon arm, a 1-cm bolus was added for 2 out of 5 fractions. To achieve a high skin dose in mastectomized patients in the proton arm, a 5-cm range shifter was applied in the treatment planning system.

For treatment planning, all patients were in supine position and immobilized using Vaclock/ Extend wing board (CIVCO MT 350) and both arms above the head. Computed tomography (CT) simulation was performed with a 3-mm slice thickness from the level of the larynx to the upper abdomen. CT simulation with either deep inspiration technique (DIBH) or free breath (FB) could be used according to the physician’s preference. CT images were transferred to Eclipse Planning System version 16.1 (Varian Medical system, Palo Alto, CA). The Clinical Target Volume (CTV) and Planning Target Volume (PTV) were delineated following the RADCOMP breast atlas v.3 guideline [13] (Appendix 2). Daily positioning was achieved based on daily cone beam computed tomography (CBCT). The treatment plan was optimized to achieve the following PTV dose distribution and normal organ dose constraints as reference from our previous dosimetric study [14].

For proton arm, all the patients were treated with intensity modulated radiation therapy (IMPT) technique. The IMPT plans were generated with two fields: an anteroposterior (AP) field (0°) and an anterior oblique field with a gantry angle ranging from (+/−) 30° to 45° depending on the beam direction to be enface as possible to the breast or chest wall. Modifications to the beam dosimetric cover at all depths were made using a 5-cm range shifter. The Nonlinear Universal Proton Optimizer (V16.1) was used to optimize dose distribution. The Proton Convolution Superposition algorithm (V16.1) with a grid size of 2.5 mm and a constant relative biological effectiveness RBE of 1.1 was used to calculate the final dose. Robust optimization with a 5-mm setup uncertainty and a 3.5% range uncertainty was employed to take uncertainties into account and enhance target coverage and organs at risk sparing. All of the patients received treatment using a 6MV VMAT approach for photon planning. The right and left breasts' VMAT plans included four arcs with gantry angles of 240° to 50° and 135° to 310°, respectively. Due to the limitation of the greatest left span of MLCs in the X-direction jaw of Varian linear accelerators, which is only 16 cm, the collimator rotated 90° for two arcs, splitting the jaw to cover the upper part and lower part of PTV, and increasing the coverage of dose in the PTV [15].

### Assessments

All patients were evaluated and had physical examinations by researchers at the beginning and weekly during radiotherapy, then at 1st week, 2nd week, 1.5 months, and 3 months after completing radiotherapy. Subsequently, assessments were scheduled every 3 months until 1 year, every 6 months until 5 years, and then annually. Acute skin toxicities were graded using the standard CTCAE version 5.0 (Appendix 3) and were assessed weekly during radiotherapy, then at 1st week, 2nd week, 1.5 months, and 3 months after radiotherapy. To evaluate the cosmetic outcome of the breast and patient satisfaction, all patients were given a questionnaire with a four-point Harvard/NSABP/RTOG cosmesis criteria scale and a four-point Likert-type scale at the end of radiotherapy, at 1.5 months after radiotherapy, and at 3 months after radiotherapy (Appendix 4).

### Objectives

The primary outcome was acute skin toxicities focused on radiation dermatitis, defined as the worst grade that collected in each assessment within 3 months after radiotherapy. Secondary endpoints included locoregional recurrence (LRR), defined as recurrence of the disease at the ipsilateral breast or regional lymph nodes (supraclavicular, axilla and internal mammary lymph nodes), cosmetic outcome and satisfaction scores from patients and other toxicities such as skin infection, pain, pneumonitis, esophagitis and fatigue.

### Statistical analysis

The objective of this study was to compare acute skin toxicity rate between proton and photon beam radiotherapy with a different rate of 30% at a significance level of 0.05 and a power of 90%. It gave a calculation result of 112 in total sample size and plus 20% to drop out so, a total of 140 patients was needed. The interim analysis was planned when 50% of patients were included to investigate the toxicity of the method. The primary purpose of this analysis was to assess the toxicity profile of the treatment arms.

The acute skin toxicities rate, good cosmetic outcome and satisfaction scores from patients were calculated by the binomial distribution and calculate the difference between proton beam radiotherapy and the photon beam with 95% confidence interval (95%CI) using two independent proportion Z-test. The difference in continuous data between two groups were calculated using an independent t-test. The difference in categorical data between groups were estimated using a Chi-square test or Fisher exact test. Statistical analysis was performed using STATA version 15.1. A *P*-value of ˂0.05 was considered statistically significant.

## RESULTS

### Patient population

**Fig. 1:**
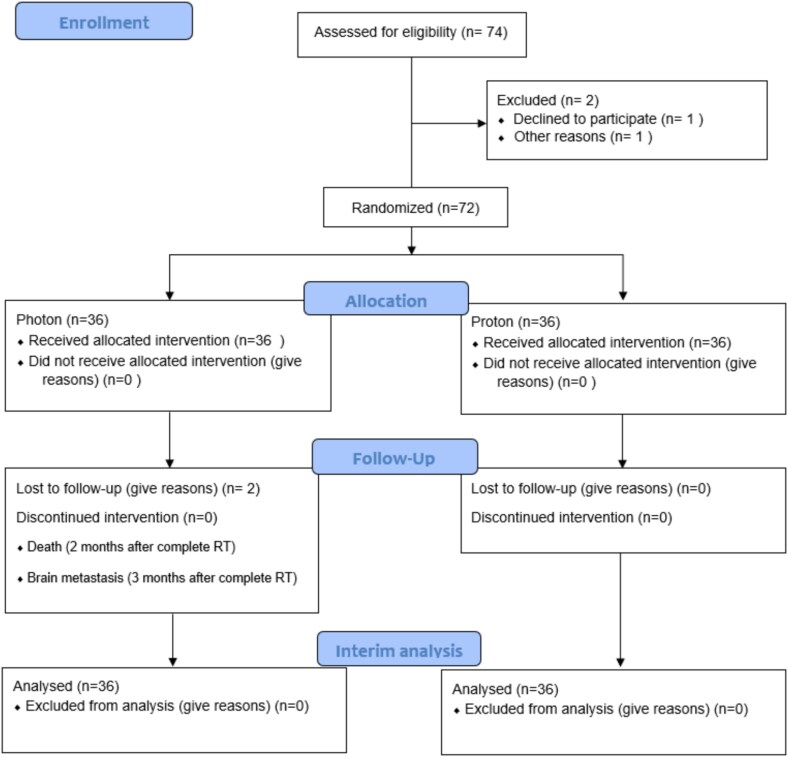
Flow diagram of the interim analysis for a randomized controlled phase II clinical trial comparing proton versus photon ultrahypofractionated radiotherapy in non-metastatic breast cancer patients. The diagram shows the number of patients assessed for eligibility, excluded, randomized into proton and photon radiotherapy groups. It also outlines reasons for exclusion and loss to follow-up during the study period.

Seventy-two patients (36 in the proton arm and 36 in the photon arm) out of a total of 140 patients who met the inclusion criteria were included in this initial analysis. Recruitment for this analysis began in August 2021, when the HRH Princess Maha Chakri Sirindhorn Proton Center at Chulalongkorn Hospital was officially opened, and the last patient was recruited in July 2022 (Fig.  1). The baseline characteristics were well-balanced between the two groups, as shown in Table 1. The mean age was 55.3 years (range 33–90), and over half of the patients had undergone breast-conserving surgery (69.44% in the proton arm, 63.89% in the photon arm). Most patients received combination chemotherapy, both neoadjuvant therapy (22.22% in the proton arm, 13.89% in the photon arm) and adjuvant therapy (55.56% in the proton arm, 52.78% in the photon arm). Two clinical T4 patients in the photon arm received neoadjuvant chemotherapy. Approximately 50% of patients in both arms received a tumor bed boost with the SIB technique. Most of the patients received radiation to the chest wall/whole breast (CW/WB) with or without the supraclavicular (SPC) region. Radiotherapy treatment details for each subgroup are presented in Appendices 5–8.

**Table 1:** Characteristics of two groups of radiotherapy in ultrahypofractionated dose

Patients’ characteristics		Proton radiotherapy (*n* = 36)	Photon radiotherapy (*n* = 36)
Ages	Mean (SD)	55.38	55.31
**ECOG**			
ECOG 0	N (%)	30 (83.33%)	32 (88.89%)
ECOG 1	N (%)	6 (16.67%)	3 (8.33%)
ECOG 2	N (%)	0 (0%)	1 (2.78%)
**Type of surgery**			
-Mastectomy	N (%)	11 (30.56%)	13 (36.11%)
Breast reconstruction	N (%)	0/11	3/13
-Breast-conserving		25 (69.44%)	23 (63.89%)
**Sites**			
-Left	N (%)	22 (61.11%)	16 (44.44%)
-Right	N (%)	14 (38.89%)	20 (55.56%)
**Pathologic T stage**		***N* = 28**	***N* = 31**
(In surgery first patients; *N* = 28, 31)	N (%)	13 (36.11%)	17 (47.22%)
T1	N (%)	15 (41.67%)	12 (33.33%)
T2	N (%)	0 (0%)	2 (5.56%)
T3	N (%)	0 (0%)	0 (0%)
T4			
**Clinical T stage**		***N* = 8**	***N* = 5**
(In neoadjuvant chemotherapy patients; *N* = 8, 5)	N (%)	0 (0%)	1 (2.78%)
T1	N (%)	6 (16.67%)	1 (2.78%)
T2	N (%)	2 (5.56%)	1 (2.78%)
T3	N (%)	0 (0%)	2 (5.56%)
T4			
**Pathologic N stage**		***N* = 28**	***N* = 31**
(In surgery first patients; *N* = 28, 31)	N (%)	13 (36.11%)	14 (38.89%)
N0	N (%)	10 (27.78%)	14 (38.89%)
N1	N (%)	4 (11.11%)	3 (8.33%)
N2	N (%)	1 (2.78%)	0 (0%)
N3			
**Clinical N stage**		***N* = 8**	***N* = 5**
(In neoadjuvant chemotherapy patients; *N* = 8, 5)	N (%)	1 (2.78%)	1 (2.78%)
N0	N (%)	6 (16.67%)	1 (2.78%)
N1	N (%)	1 (2.78%)	2 (5.56%)
N2	N (%)	0 (0%)	1 (2.78%)
N3			
**Stage groups**			
IA	N (%)	8 (22%)	11 (30.6%)
IB	N (%)	0 (0%)	0 (0%)
IIA	N (%)	11 (30.6%)	10 (27.8%)
IIB	N (%)	8 (22%)	9 (25%)
IIIA	N (%)	8 (22%)	3 (8.3%)
IIIB	N (%)	0 (0%)	2 (5.6%)
IIIC	N (%)	1 (2.8%)	1 (2.8%)
**Histology grade**			
I	N (%)	1 (2.78%)	7 (19.44%)
II	N (%)	23 (63.89%)	17 (47.22%)
III	N (%)	12 (33.33%)	12 (33.33%)
**Histology type**			
-IDC	N (%)	31 (86.11%)	32 (88.89%)
-ILC	N (%)	0 (0%)	0 (0%)
-Mucinous carcinoma	N (%)	0 (0%)	2 (5.55%)
-Other	N (%)	5 (13.89%)	2 (5.55%)
**Closest Margin**			
- < 1 mm	N (%)	8 (22.22%)	6 (16.67%)
- 1–2 mm	N (%)	3 (8.33%)	6 (16.67%)
- > 2 mm	N (%)	18 (50%)	19 (52.78%)
-Free but unspecified margin	N (%)	7 (19.44%)	5 (13.89%)
**IHC**			
-Hormone receptor +	N (%)	23 (63.89%)	27 (75%)
-HER-2 overexpression	N (%)	5 (13.89%)	4 (11.11%)
-Triple positive	N (%)	2 (5.56%)	2 (5.56%)
-Triple negative	N (%)	6 (16.67%)	3 (8.33%)
**Menopausal status**			
-Premenopausal	N (%)	14 (38.89%)	15 (41.67%)
-Postmenopausal	N (%)	22 (61.11%)	21 (58.33%)
Chemotherapy (CMT)			
-Neoadjuvant CMT	N (%)	8 (22.22%)	5 (13.89%)
-Adjuvant CMT	N (%)	20 (55.56%)	19 (52.78%)
-No CMT	N (%)	8 (22.22%)	12 (33.33%)
**Hormonal therapy**			
-Yes	N (%)	25 (69.44%)	29 (80.56%)
-No	N (%)	11 (30.56%)	7 (19.44%)
**Targeted therapy**			
-Yes	N (%)	7 (19.44%)	6 (16.67%)
-No	N (%)	29 (80.56%)	30 (83.33%)
**RT prescribed dose**			
-31 Gy/5 Fx	N (%)	19 (52.78%)	18 (50%)
-26 Gy/5 Fx	N (%)	17 (47.22%)	18 (50%)
**RT field**			
-CW/WB only	N (%)	14 (38.89%)	11 (30.56%)
-CW/WB + SPC	N (%)	12 (33.33%)	14 (38.89%)
-CW/WB + SPC + Ax	N (%)	4 (11.11%)	4 (11.11%)
-CW/WB + SPC + Ax+IMN	N (%)	6 (16.67%)	7 (19.44%)

Abbreviations: IDC – invasive ductal carcinoma, ILC – invasive lobular carcinoma, Gy – Gray, Fx – Fraction, CW – Chest wall, WB – Whole breast, SPC – supraclavicular, Ax – Axilla, IMN – internal mammary lymph node.

### Acute toxicities

The toxicities of patients, as measured by the CTCAE v.5 during and after RT up to 3 months, were recorded as the highest grade. The primary outcome, the highest recorded grade of radiation dermatitis, was significantly higher in patients treated with protons (*P* = 0.01, Table 2). Only one patient in the proton arm had grade 2 dermatitis at 2 weeks after the complete RT session, which resolved to grade 1 skin dermatitis at the next follow-up. No patient experienced grade 3, 4 or 5 radiation dermatitis. Patients were categorized into groups that experienced radiation dermatitis toxicity versus no radiation dermatitis due to the absence of grade 3 and above toxicities. The incidence of acute radiation dermatitis was significantly higher in the proton arm versus the photon arm, showing a difference of 22.2% (*P* = 0.006, Table 3). Other acute toxicities were mild, with a range of gr 0 to 2, except for skin infection. One patient in the proton arm experienced grade 3 skin infection, which resolved after intravenous antibiotics. Other acute toxicities were mild and showed no significant difference between arms (Table 3). A univariate analysis found no significant association between dermatitis and factors such as SIB boost, radiotherapy volume or type of breast surgery (Table 4). In the proton arm, the dermatitis became more pronounced during the second week of radiation therapy and remained noticeable during the first 1–2 weeks post-radiation, after which it began to improve. In contrast, in the photon arm, dermatitis became more noticeable around the second week after starting radiation therapy. These patterns are clearly illustrated in the graph included in the Appendix 9.

**Table 2:** Comparison of acute toxicities by group

	Total (*N* = 72)	Proton (*N* = 36)	Photon (*N* = 36)	*P*-value
**Dermatitis toxicity**				0.01
• Grade 0	10 (13.9)	1 (2.8)	9 (25)	
• Grade 1	61 (84.7)	34 (94.4)	27 (75)	
• Grade 2	1 (1.4)	1 (2.8)	0 (0)	
**Skin infection**				0.31
• Grade 0	71 (98.6)	35 (97.2)	36 (100)	
• Grade3	1 (1.4)	1 (2.8)	0 (0)	
**Breast pain**				0.34
• Grade 0	60 (83.3)	32 (88.9)	28 (77.8)	
• Grade 1	12 (16.7)	4 (11.1)	8 (22.2)	
**Esophagitis**				0.11
• Grade 0	52 (72.2)	25 (69.4)	27 (75)	
• Grade 1	17 (23.6)	11 (30.6)	6 (16.7)	
• Grade 2	3 (4.2)	0	3 (8.3)	
**Fatigue**				0.77
• Grade 0	57 (79.2)	28 (77.8)	29 (80.6)	
• Grade 1	15 (20.8)	8 (22.2)	7 (19.4)	

Data present n (%), *P*-value from chi-square test and or fisher exact test.

**Table 3:** Primary outcome; incidence of radiation dermatitis

	Proton (*N* = 36)		Photon (*N* = 36)		% Different (95%CI)	*P*-value
	*n*	% (95%CI)	*n*	% (95%CI)		
**Incidence of dermatitis toxicity**	35	97.2% (85.5–100)	27	75% (57.8–87.9)	22.2 (7.1–37.4)	0.006

**Table 4:** Univariate analysis of factors affecting radiation dermatitis

	Univariable	
	OR (95%CI)	*P*-value
Type of Surgery type: BCS vs MRM	2.26 (0.59–8.75)	0.236
With Boost vs no boost	1.82 (0.47–7.10)	0.388
With regional LNs vs without regional LNs	0.78 (018–3.31)	0.736

### Cosmetic outcome and satisfaction scores

Details of the worst patient-rated outcomes and satisfaction scores are presented in Table 5. Patients treated with photon reported a higher proportion of excellent cosmetic outcomes (41.7% vs 13.9%, *P* = 0.007). Patients in photon arm rated ‘very satisfied’ in a higher proportion than proton arm (80.6% vs 58.3%, *P* = 0.04) (Table 5). A photo example from one patient who rated their cosmetic outcome as excellent is included in the Appendix 10. Furthermore, as time passed, the cosmetic outcomes and satisfaction scores decreased, as illustrated in the graph provided in the Appendix 11.

**Table 5:** Cosmetic outcome and satisfaction scores by group

	Total (*N* = 72)	Proton (*N* = 36)	Photon (*N* = 36)	*P*-value
Cosmetic outcome, n (%)				0.007
• 2 Fair	3(4.2)	3 (8.3)	0 (0)	
• 3 Good	49 (68.1)	28 (77.8)	21 (58.3)	
• 4 Excellent	20 (27.8)	5 (13.9)	15 (41.7)	
Satisfaction score, n (%)				0.04
• 3 Quite satisfied	22 (30.6)	15 (41.7)	7 (19.4)	
• 4 Very satisfied	50 (69.4)	21 (58.3)	29 (80.6)	

Data present n (%), *P*-value from chi-square test.

Overall, for cosmetic outcome and satisfaction scores were used as mode statistics for all visit.

### Locoregional recurrence/distance metastasis

At a median follow-up of 9 months (IQR: 6–11), no locoregional recurrence was found in either arm. Two patients in the photon arm had distant metastases at 1 and 3 months, respectively. Both received mastectomy and systemic treatment from other hospitals before being referred for radiotherapy at our hospital. One patient in the photon arm had bone and liver metastases. She was initially classified as having clinical T4, N2 and M0 disease. After neoadjuvant chemotherapy and mastectomy, the pathological stage showed ypT4N2M0 with extra-nodal extension. Immunohistochemistry showed hormone sensitivity (ER-positive, PR-negative, HER2-negative). Liver and bone metastases were detected while she was receiving hormonal treatment at 1 month after completing radiotherapy. She passed away 2 months after completing radiotherapy. The other patient developed brain metastases 3 months after completing radiotherapy. She was initially classified as having clinical T3, N3 and M0 disease. Neoadjuvant chemotherapy followed by mastectomy was performed. Her pathological stage was ypT1N1M0 with HER2 overexpression. She is still alive and receiving systemic treatment from a medical oncologist. No distant metastasis failures were found in the proton arm.

## DISCUSSION

Our previous dosimetric study demonstrated the dosimetric advantages of proton therapy in sparing the heart and lungs compared to photon therapy [14]. This randomized controlled trial is the first to compare acute skin toxicity between proton radiotherapy and photon beam radiotherapy using ultrahypofractionation in non-metastatic breast cancer. In this interim analysis with 72 patients out of the total of 140 patients, mild (grade 1–2) radiation dermatitis was found to be significantly higher in the proton arm compared to the photon arm. The majority of radiation dermatitis cases were grade 1, except for one patient in the proton arm who experienced grade 2 radiation dermatitis. We contend that this higher incidence in the proton arm is a multifactorial result of physical beam properties, technical requirements, and the specific anatomical characteristics of our cohort. Similarly, patient-reported excellent cosmetic outcomes and a ‘very satisfied’ satisfaction score were higher in the photon arm than in the proton arm. At a median follow-up of 9 months, no locoregional recurrence was found in either arm.

The mildness of the acute radiation dermatitis in the proton arm using the 5-fraction regimen (radiation dermatitis grade 1: 94.4%, grade 2: 2.8%) compared to the previously reported 25-fractions regimen [12] (radiation dermatitis grade 1: 30.8%, grade 2: 64.1%, grade 3: 5.1%) was associated with the lower biological effective dose (BED) of ultrahypofractionation compared to conventional fractionation. In our cohort of primarily Asian women, the smaller breast volumes resulted in the tumor bed boost volume being situated in proximity to the skin rind. Unlike photon therapy which uses a natural buildup effect to spare the surface, proton therapy requires a range shifter that creates a uniform dose extending to the edge of the skin. Consequently, ensuring full target coverage of these superficial boost volumes was a higher clinical priority than skin sparing. Additionally, a higher proportion of mastectomies were performed in DeCecaris’s study compared to ours (mastectomy in proton arm: 53.8% vs. 30.56%). Compared to the previous 5-fraction regimen in the FAST Forward trial [16] (radiation dermatitis grade 1: 58%, grade 2: 36%), our study showed a higher rate of grade 1 acute radiation dermatitis in the photon arm (radiation dermatitis grade 1: 75%) but lower grade 2 acute radiation dermatitis. The difference in treatment delivered as an every-day regimen in the FAST Forward trial (5 fractions per week) and every other day in our study may be associated with less acute toxicity.

The technical discrepancy in bolus effect also contributed to these findings. A higher rate of acute radiation dermatitis in our study between the proton and photon arms may be due to the difference in radiation technique in chest wall irradiation. In the proton arm, a 5-cm range shifter was applied in every fraction for both breast-conserving surgery and chest wall patients, while a 1-cm bolus was applied in only 2 fractions of treatment in the photon arm. Therefore, the skin dose in the proton arm was higher than in the photon arm in mastectomized patients. The only patient who experienced grade 2 radiation dermatitis and the only grade 3 skin infection in the proton arm was also a mastectomy patient. Due to this observation, caution should be applied in patients who receive chest wall irradiation. However, this is an interim analysis with only ~50% of expected accrual, of which only 11 mastectomy patients were in the proton arm.

Our study has some limitations. The total number of patients in this interim analysis is too small to interpret the difference in the primary outcome, especially in mastectomized patients, which had only 11 and 13 patients in the proton and photon arms, respectively. Second, we observed only mild acute radiation dermatitis occurred in both arms, and no locoregional recurrence was found. The other limitation was the short follow-up time of 9 months, so it cannot be concluded about locoregional control and late toxicities of a 5-fraction regimen, which needs further follow-up to assess long-term cosmesis and late tissue effects.

## CONCLUSION

This interim analysis demonstrated a significantly higher incidence of acute radiation dermatitis in the proton arm of non-metastatic breast cancer patients treated with an ultrahypofractionated dose. Although the observed radiation dermatitis was only mild (grade 1–2), patients in the photon arm reported superior cosmetic outcomes and higher satisfaction scores than those in the proton arm. Ultrahypofractionation with proton therapy, while providing normal organ sparing benefits, was associated with tolerable acute toxicities. Further patient accrual and long-term follow-up are required.

## Supplementary Material

rrag037_Supplements

## Clinical trial registration number

The study protocol was approved by the Research Ethics Committee of the King Chulalongkorn Memorial Hospital (IRB number 017/64).
