# Supplementary material for: Acute skin toxicity and cosmesis outcome in non-metastatic breast cancer patients treated with ultrahypofractionated radiotherapy: a randomized controlled phase II clinical trial comparing proton versus photon radiotherapy
Source: J Radiat Res. 2026 May 25;67(4):609–17. doi: 10.1093/jrr/rrag037 (PMC13400564; doi:10.1093/jrr/rrag037)
Supplement: rrag037_Supplements [file rrag037_supplements.docx]

Acute skin toxicity and cosmesis outcome in non-metastatic breast cancer patients treated with ultrahypofractionated radiotherapy: a randomized controlled phase II clinical trial comparing proton versus photon radiotherapy

**Supplement**

**Appendix 1.** Comparison of biological effective dose and equivalent dose to 2 Gy/fraction (conventional dose) of different radiation regimens.

| Regimen | | Treated volume | BED (Gy3) | BED  (Gy4) | BED  (Gy10) | EQD2(Gy)  α/β=3 | EQD2(Gy)  α/β=4 | EQD2(Gy) α/β=10 |
| --- | --- | --- | --- | --- | --- | --- | --- | --- |
| Conventional dose | 2 Gy x 25 fractions = 50 Gy | Whole breast | 83.33 | 75 | 60 | 50 | 50 | 50 |
| Conventional dose | 2 Gy x 33 fractions = 66 Gy | Tumor bed boost | 110 | 99 | 79.2 | 66 | 66 | 66 |
| Ultrahypofraction dose | 5.2 Gy x 5 fractions = 26 Gy | Whole breast | 71.07 | 59.8 | 39.52 | 42.64 | 39.87 | 32.93 |
| Ultrahypofraction dose | 6.2 Gy x 5 fractions = 31 Gy | Tumor bed boost | 95.07 | 79.05 | 50.22 | 57.04 | 52.7 | 41.85 |

**Appendix 2**

**Target delineation**

Breast and chest wall contour: Anatomical Boundaries

|  | Cranial | Caudal | Anterior | Posterior | Lateral | Medial |
| --- | --- | --- | --- | --- | --- | --- |
| Chest wall/  Breast | Caudal border of the clavicle head | Approximately 1-2 cm below breast tissue /prior breast tissue | Skin | Up to but not including ribs | Mid axillary line | Sternum |

Note: In case with not including axilla level I&II, chest wall may extend more posterior

Regional nodal contours: Anatomical boundaries

|  | Cranial | Caudal | Anterior | Posterior | Lateral | Medial |
| --- | --- | --- | --- | --- | --- | --- |
| Supra clavicular | Caudal to the cricoid cartilage | Connect to Internal  Mammary node (IMN) (includes subclavian vein) | Dorsal surface of Sternocleidomastoid (SCM) muscle (m.), clavicle or strap muscles | Anterior & medial aspect of the scalene m., levator scapulae, posterior edge of SCM & vascular region (not more than pleura) | lateral edge of SCM, clavicle & connecting to level III axilla | Medial edge of carotid artery |
| Posterior Neck | Caudal to the cricoid cartilage | Obliteration of fat space | Supraclavicular volume | Trapezius | Platysma, level II or III scapular | The longus coli |
| Axilla  level I | Axillary vessels at lateral edge of Pec. Minor m.& below humeral head | Pectoralis (Pec.) major muscle insert into ribs | Posterior surface of Pec. Major. m. or skin | Anterior surface of subscapularis m. and latissimus dorsi | Lat. Dorsi m., at line connecting latissimus dorsi and deltoid or up to skin | Lateral border of Pec.  Minor m. or level II |
| Axilla  level II | At or caudal to Pec Minor m. insertion on coracoid | Obliteration of fat space between Pec. Major m. and Pec. Minor m. or chest wall. | Posterior surface Pec. Major m. | Chest wall | Level I or lateral border of Pec Minor m. | Medial border of Pec Minor or level III |
| Axilla  level III | Caudal to Pec. Minor m. insert on Cricoid | Obliteration of fat space between Pec. Major m. and chest wall. | Posterior surface Pec. Major m. | Chest wall | Medial border of Pec. Minor m. or level II | Obliteration of fat space and the supraclavicular volume |
| Internal  mammary | Inferior supraclavicular volume or caudal to head of clavicle | Cranial aspect of the 4 rib | Posterior chest wall | Posterior to pleura (include fat but not lung) | Lateral to include any visible fat | Sternum |

For Boost tumor bed in both arm, CTV = Tumor bed, PTV = Tumor bed + 1 cm, CTV tumor bed: outlined by contouring around the implanted markers or any changes in the surrounding tissue architecture based on location and size of tumor before surgery.

**Appendix 3**

**CTCAE version 5**

|  | Result | | | | |
| --- | --- | --- | --- | --- | --- |
| Side effect grading | 1 | 2 | 3 | 4 | 5 |
| Dermatitis radiation | Faint erythema or dry desquamation | Moderate to brisk erythema; patchy moist desquamation, mostly confined to skin folds and creases; moderate edema. | Moist desquamation in areas other than skin folds and creases; bleeding induced by minor trauma or abrasion. | Life threatening consequences; skin necrosis or ulceration of full thickness dermis; spontaneous bleeding from involved site; skin graft indicated. | Death |
| Skin infection | Localized, local intervention indicated | Oral intervention indicated (e.g., antibiotic, antifungal, or antiviral) | IV antibiotic, antifungal, or antiviral intervention indicated; invasive intervention indicated | Life-threatening consequences; urgent intervention indicated | Death |
| Fatigue | Fatigue relieved by rest | Fatigue not relieved by rest; limiting instrumental ADL | Fatigue not relieved by rest, limiting self-care ADL | - | - |
| Esophagitis | Asymptomatic; clinical or diagnostic observations only; intervention not indicated | Symptomatic; altered eating/swallowing; oral supplements indicated | Severely altered eating/swallowing; tube feeding, TPN, or hospitalization indicated | Life-threatening consequences; urgent operative intervention indicated | Death |
| Dyspnea | Shortness of breath with moderate exertion | Shortness of breath with minimal exertion; limiting instrumental ADL | Shortness of breath at rest; limiting self-care ADL | Life-threatening consequences; urgent intervention indicated | Death |
| Nausea | Loss of appetite without alteration in eating habits | Oral intake decreased without significant weight loss, dehydration or malnutrition | Inadequate oral caloric or fluid intake; tube feeding, TPN, or hospitalization indicated | - | - |
| Tumor pain | Mild pain | Moderate pain: limiting instrumental ADL | Severe pain: limiting self-care ADL |  |  |
| Myocarditis | - | Symptoms with moderate activity or exertion | Severe with symptoms at rest or with minimal activity or exertion; intervention indicated; new onset of symptoms | Life-threatening consequences; urgent intervention indicated (e.g., continuous IV therapy or mechanical hemodynamic support) | Death |
| Pericarditis | Asymptomatic, ECG or physical findings (e.g., rub) consistent with pericarditis | Symptomatic pericarditis (e.g., chest pain) | Pericarditis with physiologic consequences (e.g., pericardial constriction) | Life-threatening consequences; urgent intervention indicated | Death |
| Pneumonitis | Asymptomatic; clinical or diagnostic observations only; intervention not indicated | Symptomatic; medical intervention indicated; limiting instrumental ADL | Severe symptoms; limiting selfcare ADL; oxygen indicated | Life-threatening respiratory compromise; urgent intervention indicated (e.g., tracheotomy or intubation) | Death |

**Appendix 4. Cosmetic outcome and satisfaction scores assessment**

4 Excellent When compared to the untreated breast, there is minimal or no difference in the size or shape of the treated breast. The way the breast feels (its texture) is the same or slightly different. There may be thickening, scar tissue, or fluid accumulation within the breast but not enough to change the appearance.

3 Good There is a slight difference in the size or shape of the treated breast as compared to the opposite breast or the original appearance of the treated breast. There may be some mild reddening or darkening of the breast. The thickening or scar tissue causes only a mild change in the shape or size.

2 Fair Obvious difference in the size and shape of the treated breast. This change involves one-quarter or less of the breast. There can be moderate thickening or scar tissue of the skin and the breast, and there may be obvious color changes.

1 Poor Marked change in the appearance of the treated breast involving more than one-quarter of the breast tissue. The skin changes may be obvious and detract from the appearance of the breast. Severe scarring and thickening of the breast, which clearly alters the appearance of the breast, may be found.

Likert-type scale

4 Very satisfied

3 Quite satisfied

2 Not quite satisfied

1 Unsatisfied

**Appendix 5. Dosimetric evaluation for Lt./Rt. chest wall with regional nodes comparing IMPT versus VMAT**

| Organ  Dose levels | | Lt. chest wall+regional nodes | | | Rt. chest wall+regional nodes | | |
| --- | --- | --- | --- | --- | --- | --- | --- |
|  |  | IMPT (N=7) | VMAT (N=5) | P value | IMPT (N=4) | VMAT (N=7) | P value |
| CTV/PTV* | Dmax(GyRBE) | 30.3 (1.5) | 29 (0.6) | 0.11 | 31.7 (0.5) | 30.8 (2.3) | 0.46 |
| mean (SD) | D90(GyRBE) | 25.5 (0.9) | 24.5 (1.2) | 0.12 | 26.4 (1) | 25.5 (1.5) | 0.37 |
|  | D95(GyRBE) | 23.8 (1.8) | 22.7 (2.4) | 0.40 | 24.7 (1.7) | 24 (2.4) | 0.62 |
| Ipsi-lung | Dmean(GyRBE) | 5.5 (1.9) | 7.9 (0.9) | 0.03 | 5.2 (1.3) | 8.9 (1.3) | 0.002 |
| mean (SD) | V5(%) | 40.5 (13.3) | 58.6 (18.2) | 0.07 | 26.4 (18.1) | 65.1 (15.1) | 0.004 |
|  | V10(%) | 22.2 (10.4) | 25.9 (3.7) | 0.47 | 13.4 (9.8) | 32.4 (6.1) | 0.003 |
|  | V20(%) | 2.8 (2.1) | 6.8 (3.2) | 0.02 | 2 (2.1) | 8.4 (3.4) | 0.008 |
| Contra-lung | Dmean(GyRBE) | 0.2  (0.1-0.3) | 3  (2.9-4.6) | 0.04 | 0.1  (0-0.3) | 3.2  (2.5-4.2) | 0.008 |
| Median (IQR) | V5(%) | 0.1  (0-0.8) | 16.6  (15.9-31.2) | 0.004 | 0  (0-0) | 14.6  (13.6-32.6) | 0.007 |
|  | V10(%) | 0  (0-0.1) | 2.6  (2.3-2.6) | 0.005 | 0  (0-0) | 1.2  (0.5-2.9) | 0.02 |
| Heart | Dmax(GyRBE) | 21.4  (8.1-26.1) | 22.5  (19.8-25.2) | 0.81 | 14.8  (10.1-18.8) | 16  (14.3-20.7) | 0.45 |
|  | Dmean(GyRBE) | 0.5  (0.2-2) | 4.2  (4.1-4.3) | 0.005 | 0.2  (0.1-0.3) | 3.1  (2.6-4) | 0.01 |
| Median (IQR) | V5(%) | 2.7  (0.3-13.4) | 23.8  (22.4-27.3) | 0.01 | 1  (0.5-1.8) | 11.7  (7.1-26.6) | 0.008 |
|  | V10(%) | 0.6  (0-5.7) | 7.3  (7.2-7.9) | 0.06 | 0.1  (0-0.3) | 2.3  (0.4-5) | 0.04 |
|  | V20(%) | 0 (0-0.5) | 0.1 (0-0.2) | 0.79 | 0 (0-0) | 0 (0-0) | NA |
| Esophagus | Dmax(GyRBE) | 22.9 (4.2) | 24.5 (2.8) | 0.47 | 16.5 (2.3) | 18.6 (4.3) | 0.39 |
| mean (SD) | Dmean(GyRBE) | 2.8 (1) | 6.2 (1.1) | <0.001 | 1.7 (0.9) | 5.6 (1.3) | 0.001 |
| Thyroid | Dmax(GyRBE) | 25.4 (2.6) | 27.6 (1) | 0.10 | 28.1 (0.2) | 28.6 (0.7) | 0.17 |
| mean (SD) | Dmean(GyRBE) | 8.9 (3.6) | 15.3 (2.1) | 0.006 | 11 (2.5) | 15.4 (3.4) | 0.05 |
| Brachial plexus, mean (SD) | Dmax(0.5cm3)  (GyRBE) | 26.6 (1.5) | 26.7 (0.4) | 0.85 | 28.5 (0.7) | 27.5 (1.6) | 0.26 |

Compare mean using two independent sample t-test, compare median using Wilcoxon rank sum test, *CTV for proton, PTV for VMAT

**Appendix 6. Dosimetric evaluation for Lt./Rt. whole breast irradiation comparing IMPT versus VMAT for with regional nodes**

Compare mean using two independent sample t-test, compare median using Wilcoxon rank sum test, *CTV for proton, PTV for VMAT

| Organ  Dose levels | | Lt. whole breast +regional nodes | | | Rt. whole breast +regional nodes | | |
| --- | --- | --- | --- | --- | --- | --- | --- |
|  |  | IMPT (N=8) | VMAT (N=6) | P value | IMPT (N=3) | VMAT (N=7) | P value |
| CTV/PTV* | Dmax(GyRBE) | 32.8 (3.3) | 33.1 (1.1) | 0.82 | 31.7 (4.8) | 33.8 (2.5) | 0.36 |
| mean (SD) | D90(GyRBE) | 27.1 (1) | 26.2 (1.1) | 0.14 | 24.1 (3.5) | 24.9 (2.1) | 0.64 |
|  | D95(GyRBE) | 26.2 (1.5) | 24.8 (2.6) | 0.22 | 22.2 (3.9) | 23 (2.9) | 0.74 |
| Ipsi-lung | Dmean(GyRBE) | 4 (1.4) | 6.8 (0.7) | 0.001 | 3.3 (0.5) | 9.1 (2.1) | 0.001 |
| mean (SD) | V5(%) | 27.3 (14.6) | 47.2 (5.9) | 0.009 | 24.1 (3.2) | 66 (12.6) | 0.001 |
|  | V10(%) | 13 (9.2) | 21.3 (3.5) | 0.06 | 11.5 (2.8) | 34.2 (13.8) | 0.03 |
| Median (IQR) | V20(%) | 0.8 (0.2-1.5) | 3.8 (3.5-7.6) | 0.003 | 0.7 (0.1-2.2) | 7.7 (6.1-9.4) | 0.09 |
| Contra-lung | Dmean(GyRBE) | 0.2  (0.1-0.3) | 3.3  (2.7-3.6) | 0.002 | 0  (0-0) | 3.7  (2.9-3.8) | 0.02 |
| Median (IQR) | V5(%) | 0.6  (0-1.1) | 18.3  (13-24) | 0.002 | 0  (0-0) | 23.7  (14.4-29.8) | 0.01 |
|  | V10(%) | 0.1 (0-0.2) | 0.4 (0.3-3.6) | 0.04 | 0 (0-0) | 1.4 (0.2-1.9) | 0.03 |
| Heart | Dmax(GyRBE) | 20.7  (16.3-23.9) | 14.4  (11.2-20.9) | 0.37 | 2.6  (2.1-4.5) | 20.8  (15.6-24.2) | 0.02 |
| Median (IQR) | Dmean(GyRBE) | 0.4 (0.3-0.5) | 2.7 (2.4-2.9) | 0.002 | 0 (0-0.1) | 3.6 (3-4.4) | 0.02 |
|  | V5(%) | 1.9  (1-2.8) | 7.9  (7-11.7) | 0.002 | 0  (0-0) | 16.7  (11.4-27.4) | 0.02 |
|  | V10(%) | 0.5 (0.2-0.8) | 0.2 (0-3.6) | 0.65 | 0 (0-0) | 2.1 (0.5-5.9) | 0.03 |
|  | V20(%) | 0 (0-0) | 0 (0-0) | 0.25 | 0 (0-0) | 0 (0-0.1) | 0.33 |
| Esophagus | Dmax(GyRBE) | 23.2 (1.7) | 23.3 (3.4) | 0.99 | 13.3 (1.8) | 19.5 (7.1) | 0.18 |
| mean (SD) | Dmean(GyRBE) | 2.3 (0.9) | 5.2 (1.4) | 0.001 | 0.8 (0.3) | 6.5 (2.3) | 0.003 |
| Thyroid | Dmax(GyRBE) | 26.6 (1.9) | 28.2 (1.6) | 0.13 | 23.7 (1.8) | 25.7 (7.7) | 0.68 |
| mean (SD) | Dmean(GyRBE) | 9.6 (2.8) | 11.7 (1.2) | 0.10 | 8.6 (1.9) | 13.5 (5.4) | 0.18 |
| Brachial plexus, mean (SD) | Dmax(0.5cm3)  (GyRBE) | 28  (1) | 27.1  (1) | 0.12 | 26  (3.9) | 24.7  (5.8) | 0.73 |

**Appendix 7. Dosimetric evaluation for Lt./Rt. whole breast irradiation comparing IMPT versus VMAT**

| Organ  Dose levels | | Lt. whole breast | | | Rt. whole breast | | |
| --- | --- | --- | --- | --- | --- | --- | --- |
|  |  | IMPT (N=7) | VMAT (N= 4) | P value | IMPT (N=7) | VMAT (N=6) | P value |
| CTV/PTV* | Dmax(GyRBE) | 33.2 (1.7) | 32.2 (2.7) | 0.48 | 33.9 (1.2) | 33.8 (2.1) | 0.94 |
| mean (SD) | D90(GyRBE) | 26.2 (0.9) | 26.6 (2) | 0.67 | 26.4 (1.3) | 26.4 (1.5) | 0.96 |
|  | D95(GyRBE) | 25.4 (1.6) | 24.9 (4.1) | 0.80 | 25.2 (2.5) | 24.6 (3.2) | 0.69 |
| Ipsi-lung | Dmean(GyRBE) | 3.1 (2.1) | 6.1 (2) | 0.04 | 3 (1.1) | 6.3 (1.4) | 0.001 |
| mean (SD) | V5(%) | 20.5 (13.5) | 36.6 (12.7) | 0.08 | 16.9 (9.6) | 40.7 (16.4) | 0.008 |
| Median (IQR) | V10(%) | 9.6 (1.7-16.6) | 19.2 (12.5-26.5) | 0.09 | 5.8 (4.3-12.4) | 21.2 (14.6-23.2) | 0.02 |
| Median (IQR) | V20(%) | 0.5 (0-3) | 6.3 (2.7-11.2) | 0.04 | 0.3 (0.1-1.4) | 4.1 (2.3-8.1) | 0.04 |
| Contra-lung | Dmean(GyRBE) | 0.1 (0.1-0.1) | 2.5 (2.3-2.7) | 0.008 | 0 (0-0.2) | 2.5 (1.8-2.6) | 0.003 |
| Median (IQR) | V5(%) | 0 (0-0.1) | 11.7 (8.8-15.2) | 0.006 | 0 (0-0.1) | 7.6 (5.5-12) | 0.002 |
|  | V10(%) | 0 (0-0) | 1.3 (0.3-2) | 0.002 | 0 (0-0) | 0 (0-0) | 0.82 |
| Heart | Dmax(GyRBE) | 20.5 (11.4-24.1) | 22.3 (17.3-25.8) | 0.45 | 7.5 (6-15.1) | 9.5 (6.9-11.8) | 0.89 |
|  | Dmean(GyRBE) | 0.4 (0.2-0.8) | 3.4 (2.6-3.9) | 0.01 | 0.1 (0.1-0.3) | 2.5 (1.9-3) | 0.003 |
| Median (IQR) | V5(%) | 1.9 (0.4-5.4) | 17.8 (9.9-21.1) | 0.02 | 0 (0-1.5) | 5.4 (0.9-10.3) | 0.08 |
|  | V10(%) | 0.4 (0-2.1) | 4.1 (1.5-6.4) | 0.13 | 0 (0-0.1) | 0 (0-0.1) | 0.86 |
|  | V20(%) | 0 (0-0.2) | 0.4 (0-0.9) | 0.27 | 0 (0-0) | 0 (0-0) | NA |
| Esophagus | Dmax(GyRBE) | 0.1 (0-0.3) | 3.9 (2.2-6.5) | 0.02 | 0.1 (0.1-0.8) | 4.7 (3.9-5.8) | 0.003 |
| Median (IQR) | Dmean(GyRBE) | 0 (0-0) | 1.5 (1.1-1.9) | 0.05 | 0 (0-0) | 2.2 (1.6-2.5) | 0.002 |
| Thyroid | Dmax(GyRBE) | 0.7 (0.4-1.1) | 0.5 (0.4-0.8) | 0.56 | 0.3 (0-1.2) | 1 (0.7-4.4) | 0.12 |
| Median (IQR) | Dmean(GyRBE) | 0.1 (0.1-0.2) | 0.3 (0.2-0.7) | 0.09 | 0 (0-0.2) | 0.6 (0.4-1) | 0.003 |
| Brachial plexus, Median (IQR) | Dmax(0.5cm3)  (GyRBE) | 0.1  (0-0.1) | 0.4  (0.4-0.5) | 0.06 | 0  (0-0.3) | 0.7  (0.5-3.8) | 0.006 |

Compare mean using two independent sample t-test, compare median using Wilcoxon rank sum test, *CTV for proton, PTV for VMAT

**Appendix 8. Dosimetric evaluation for for Lt./Rt. Chest wall**

Compare mean using two independent sample t-test, compare median using Wilcoxon rank sum test, *CTV for proton, PTV for VMAT

| Organ  Dose levels | | Lt. Chest wall | | | Rt. Chest wall | | |
| --- | --- | --- | --- | --- | --- | --- | --- |
|  |  | IMPT (N=0) | VMAT (N= 1) | P value | IMPT (N=0) | VMAT (N=0) | P value |
| CTV/PTV* | Dmax(GyRBE) | - | 27.97 | - | - | - | - |
| mean (SD) | D90(GyRBE) | - | 24.19 | - | - | - | - |
|  | D95(GyRBE) | - | 22.42 | - | - | - | - |
| Ipsi-lung | Dmean(GyRBE) | - | 6.32 | - | - | - | - |
| mean (SD) | V5(%) | - | 41.7 | - | - | - | - |
| Median (IQR) | V10(%) | - | 19 | - | - | - | - |
| Median (IQR) | V20(%) | - | 4.7 | - | - | - | - |
| Contra-lung | Dmean(GyRBE) | - | 2.38 | - | - | - | - |
| Median (IQR) | V5(%) | - | 10.1 | - | - | - | - |
|  | V10(%) | - | 0.1 | - | - | - | - |
| Heart | Dmax(GyRBE) | - | 20.32 | - | - | - | - |
|  | Dmean(GyRBE) | - | 2.88 | - | - | - | - |
| Median (IQR) | V5(%) | - | 12.2 | - | - | - | - |
|  | V10(%) | - | 2,4 | - | - | - | - |
|  | V20(%) | - | 0 | - | - | - | - |
| Esophagus | Dmax(GyRBE) | - | 22.42 | - | - | - | - |
| Median (IQR) | Dmean(GyRBE) | - | 3.6 | - | - | - | - |
| Thyroid | Dmax(GyRBE) | - | 26.38 | - | - | - | - |
| Median (IQR) | Dmean(GyRBE) | - | 11.31 | - | - | - | - |
| Brachial plexus, Median (IQR) | Dmax(0.5cm3)  (GyRBE) | -  - | 25.02 | - | -  - | -  - | - |

**Appendix 9. Timeline of radiation dermatitis severity (Grade 0-2) in proton vs photon arms during and after radiotherapy**

**The x-axis represents the time points during and after radiotherapy, starting from "During RT" to 3 months post-RT. The y-axis shows the percentage of patients experiencing different grades of dermatitis (Grade 0, Grade 1, and Grade 2).**

**Appendix 10. Example of excellent cosmetic outcome from a patient ( Right breast treated)**

**
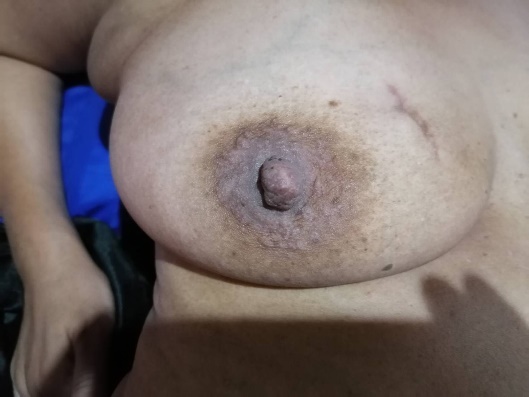

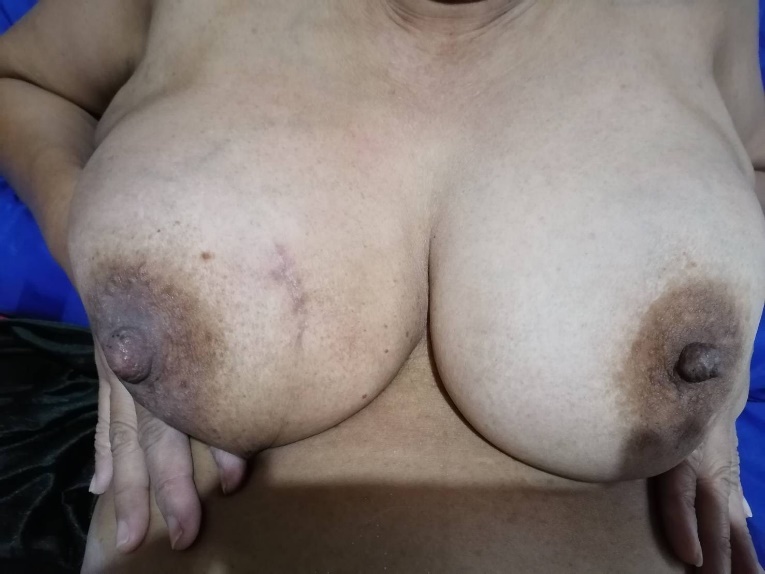
**

**Appendix 11. Changes in cosmetic outcomes and satisfaction scores over time for proton and photon arms**

**7A**

**The x-axis represents the time points during and after radiotherapy, starting from "During RT" to 1-3 months post-radiotherapy. The y-axis shows the percentage of patients with cosmetic outcomes rated as Fair, Good, or Excellent. As time passed, cosmetic outcomes gradually declined in both the proton and photon arms, with fewer patients reporting "excellent" outcomes post-radiotherapy.**

**7B**

**The x-axis represents the time points during and after radiotherapy, starting from "During RT" to 1-3 months post-radiotherapy. The y-axis shows the percentage of patients with satisfaction scores rated as 2 (neutral), 3 (quite satisfied), or 4 (very satisfied). As time progressed, patient satisfaction scores decreased slightly in both the proton and photon arms, with a lower percentage of patients rating their experience as "very satisfied" post-radiotherapy.**
